# Basal body temperature as a biomarker of reproductive state in ewes: A multi‐method comparison

**DOI:** 10.1111/avj.70112

**Published:** 2026-07-04

**Authors:** E Pérez‐García, J Nieto de la Losa, JA Abecia, J Plaza, C Palacios Riocerezo

**Affiliations:** ^1^ Faculty of Environmental and Agricultural Sciences University of Salamanca Salamanca Spain; ^2^ Institute of Research in Environmental Sciences of Aragón (IUCA), University of Zaragoza Zaragoza Spain

**Keywords:** biologger, infrared thermography, mixed‐effects model, oestrous cycle, rectal temperature, sheep

## Abstract

This study quantified body temperature dynamics throughout reproductive stages in crossbred ewes based on complementary monitoring techniques. Five ewes from a semi‐extensive farming system were included in two sub‐trials: animals were first assessed in the prepubertal stage and, subsequently, an oestrous cycle was induced by intravaginal progesterone sponges followed by eCG and prostaglandin; oestrous was confirmed by serum progesterone. Temperatures were recorded in five stages (prepubertal, pubertal, sponge, post‐sponge, oestrous) based on eight measurements: rectal (RT), subcutaneous biologger (SCT) and infrared thermography (IRT) of vulva/axilla that were obtained either as unprocessed maximum pixel or as FLIR‐processed maximum/mean. A linear mixed‐effects model that included Method, Stage and their interaction indicated strong global effects (all *p* < 0.001). Estimated marginal means revealed a clear physiological trajectory: temperatures were highest in the prepubertal stage, generally decreased from the prepubertal stage towards the treatment‐related stages, although the timing of the minimum value depended on the measurement method. Among the methods, RT yielded the highest estimated mean temperatures and the most stable measurements, SCT recorded slightly lower temperatures, vulvar FLIR‐processed metrics were able to detect stage‐related thermal changes, and axillary measures, especially unprocessed maxima, were more variable and environmentally sensitive. In general, thermal monitoring identified the expected reproductive‐stage patterns. RT and SCT provided the most stable detection of reproductive‐stage temperature changes, whereas vulvar FLIR‐processed thermography represented the most promising non‐invasive alternative. Axillary thermography was more variable and appeared to be more susceptible to environmental influences.

## Introduction

Sheep production is a cornerstone of rural sustainability, because it contributes to social, environmental and economic outcomes, particularly in disadvantaged agricultural areas.^1^ Reproductive efficiency is a primary determinant of profitability in sheep systems,^2^ yet many breeds display marked reproductive seasonality that is regulated by photoperiod, specifically, sexual inactivity in the long‐day period and short days trigger the fertile phase, signalling is strongest at high latitudes and attenuated in Mediterranean breeds.^3^, ^4^, ^5^

Detecting oestrous in ewes is challenging because external signs (e.g. vulvar oedema or erythema) are subtle and often obscured by wool. Teaser rams can aid detection but add cost and are not always effective.^6^ Fixed‐time artificial insemination (AI) protocols based on progestagens, gonadotrophins and prostaglandins offer management gains,^7^, ^8^ although concerns remain about extensive hormone use and long‐term sustainability.^9^, ^10^

Against that backdrop, thermal monitoring has emerged as a non‐invasive tool to aid reproductive management. Ovine homeothermy is controlled by coordinated central and peripheral mechanisms,^11^, ^12^ and body temperature (*T*) is influenced by hormonal milieu, oestrous phase and circadian rhythm.^13^, ^14^ Although progesterone is associated with an increase in luteal *T* in humans, the relationship is less consistent in sheep, and decreases in luteal *T* occur around ovulation or following progestagen treatments.^15^ Advances in subcutaneous biologgers and infrared thermography (IRT), including software‐based processing of regions of interest (ROI), enable high‐resolution, non‐contact assessments that are useful in attaining welfare and sustainability goals^16^, ^17^; however, there have been limited head‐to‐head comparisons of rectal temperature (RT), subcutaneous temperature (SCT) and IRT approaches, particularly contrasting processed ROI metrics *vs*. unprocessed hottest‐pixel readings across defined reproductive stages under farm conditions.

We assessed whether body *T* in ewes exhibits a systematic trajectory throughout the prepubertal‐to‐oestrous transition under commercial‐like conditions, and how accurately different measurement methods track that trajectory. Our aim was to quantify stage‐related thermal dynamics and to compare the discriminative performance of RT, SCT and IRT (vulvar and axillary windows; FLIR‐processed *vs*. unprocessed) based on a linear mixed‐effects framework that included repeated measures within animals. The results can help identify non‐invasive thermal biomarkers for improving reproductive monitoring and management.

## Material and methods

### Animals and experimental design

The study was conducted on a commercial sheep farm in Florida de Liébana, Salamanca, Spain (41.00686, −5.77519), in the north‐western Iberian Peninsula. The flock comprised 110 crossbred ewes (Castellana × Berrichon du Cher × Île de France) that were managed under a semi‐extensive system.

All procedures were performed in accordance with Spanish animal welfare regulations (RD 1201/2005) and the European Union Directive 2010/63 on the protection of animals used for scientific purposes. The Animal Experimentation Ethics Committee of the University of Salamanca approved the experimental protocol (approval number 904).

Five crossbred ewes were used in two sub‐trials. Animals entered the study at 4 months of age (30 ± 7 kg; prepubertal) and reached oestrous at around 7 months of age (55 ± 8 kg). The study was conducted in the Northern Hemisphere during the transition from summer to autumn, when day length progressively decreases following the summer solstice. In sheep, photoperiod is the principal environmental cue regulating seasonal reproductive activity. Changes in daylight duration are translated into endocrine signals through melatonin secretion by the pineal gland, influencing the hypothalamic‐pituitary‐gonadal axis and the seasonal regulation of reproduction.^18^ These photoperiodic conditions are representative of Mediterranean sheep production systems and differ from those occurring in Southern Hemisphere regions, including Australia, where seasonal patterns are reversed.

- Sub‐trial 1 (Prepubertal monitoring). Continuous thermal monitoring was performed in all five ewes in the prepubertal period. This sub‐trial was conducted from 16 July 2024 to 19 July 2024.
- Sub‐trial 2 (Oestrous induction under natural photoperiod). At approximately 7 mo, the same ewes received intravaginal progestagen‐impregnated sponges (SYNCRO‐PART®, CEVA Animal Health, Spain) that remained in place for 7 days. At sponge withdrawal, 480 IU of eCG (SYNCRO‐PART® PMSG, intramuscular route) and 2 mL/ewe of prostaglandin F2α (Prosyl®, CEVA Animal Health, Spain) were administered. Monitoring commenced 2 days before sponge insertion, continued throughout the treatment period and was maintained following sponge withdrawal and in the oestrous phase. This subtrial was conducted from 19 October 2024 to 31 October 2024.

For the analyses, we defined five physiological recording windows that correspond directly to Reproductive stage, a factor included in the mixed model (Section: Statistical analysis), as follows: (1) Prepubertal = 4 days of recording (≈4 months of age), (2) Pubertal (pre‐synchronisation) = 2 days (≈7 months, before sponge insertion), (3) Sponge = 7 days (duration of intravaginal treatment), (4) Post‐sponge (D + 1) = 1 day immediately after sponge withdrawal, (5) Possible Oestrous = 3 days following sponge removal (oestrous window).

### Temperature monitoring

#### Subcutaneous biologgers

Each ewe carried a DST milli‐HRT ACT biologger (Star Oddi, Gardabaer, Iceland; 13 × 39.5 mm, 12 g; Figure 1C) that had been programmed to record subcutaneous *T* at 5‐min intervals. In Sub‐trial 1, the devices remained implanted for 1 week. In Sub‐trial 2, sensors were implanted in advance and monitoring began 2 days before sponge insertion, continued throughout the 7‐day treatment and was maintained after sponge withdrawal, including the oestrous window.

**Figure 1:**
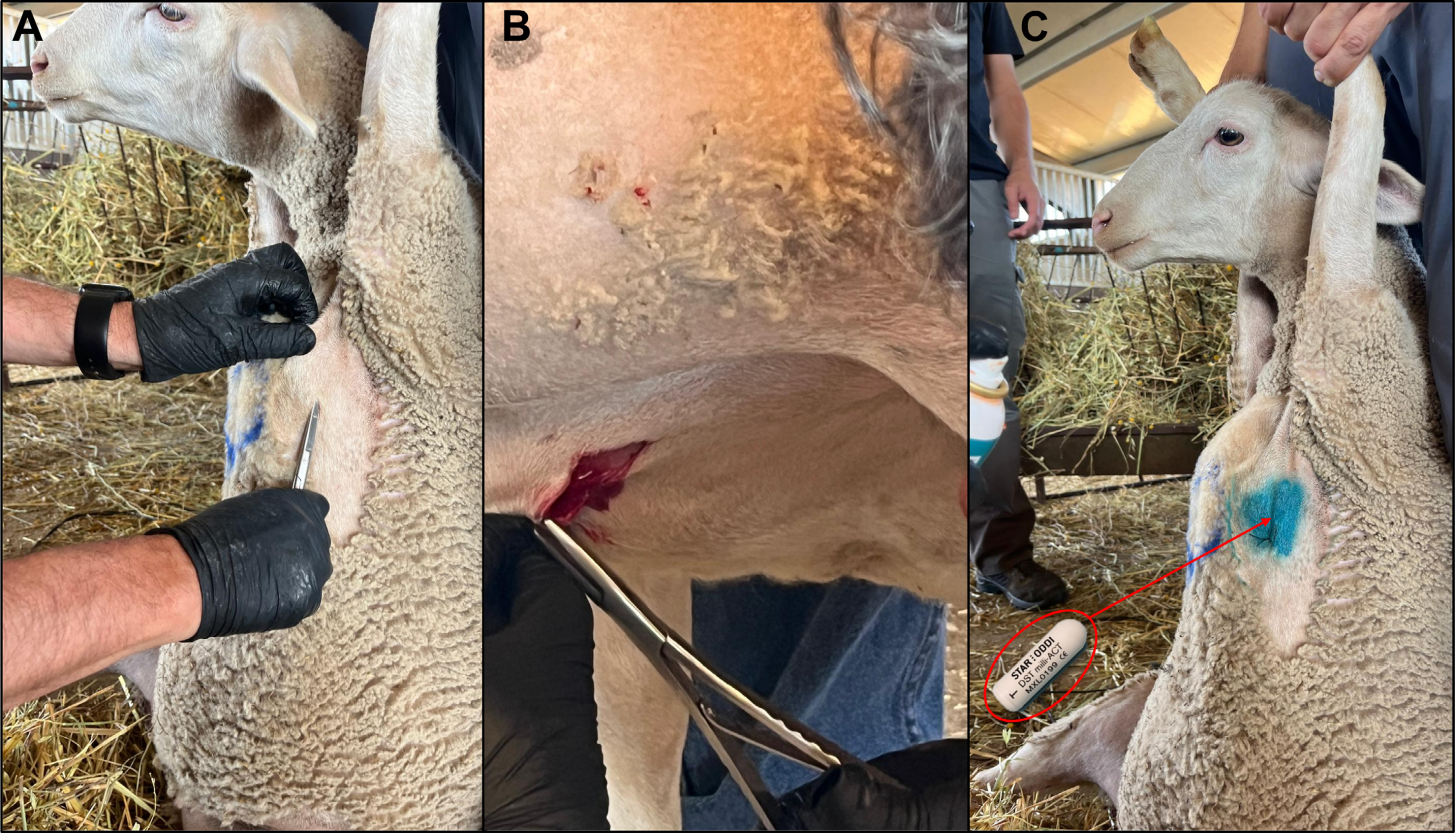
(A) Positioning of the ewe for biologger implantation, which exposed the left thoracic region; (B) Creation of the subcutaneous pocket for housing the biologger; (C) Skin closure with 2 or 3 sutures and aluminium spray protection, and details of the biologger.

Before implantation, biologgers underwent high‐level disinfection by immersion in 0.55% ortho‐phthalaldehyde (CIDEX‐OPA, Johnson & Johnson, USA) for 24 h. Implantation was performed in the left thoracic region, above the heart and aligned parallel to its axis (Figure 1A). Ewes were restrained in a handling pen, sedated with intramuscular xylazine (0.05 mg/kg; Xilagesic, Calier, Spain) and locally anaesthetised with 2 mL lidocaine hydrochloride (Anesvet, Ovejero, Spain). After aseptic preparation, a small skin incision was made and a subcutaneous pocket was created to accommodate the device (Figure 1B). The incision was closed with 2–3 sutures (surgical silk), and an aluminium spray was applied as protection (Aluspray®, Vetoquinol, Spain) (Figure 1(C)). Removal of the biologger followed the same surgical approach.

#### Infrared thermography (IRT) and digital thermometry

To maximise the number of observations and assess peripheral sites, thermographic imaging and rectal T measurements were performed within a fixed daily window (1630–1715 h) throughout the study. IRT was recorded by a handheld FLIR E54‐EST camera (Teledyne FLIR LLC, USA; 320 × 240 pixels, accuracy ±0.2°C), and thermographic images were subsequently analysed using FLIR Thermal Studio software (Teledyne FLIR LLC, Wilsonville, OR, USA). Four thermographic measurement events were performed at 15‐min intervals (1630, 1645, 1700 and 1715 h). During each session, one thermographic image of the vulvar region and one of the axillary region were captured. The images focused on two regions of interest (ROIs): the vulva and the left axilla (external insertion of the forelimb). For axillary thermographic images, the forelimb was gently raised to expose the axillary region, while for vulvar thermographic images, the tail was gently raised to allow unobstructed visualisation of the vulvar area. All images were acquired indoors, with an emissivity = 0.98, at a distance of approximately 0.5 m from the animals, and with autofocus enabled. During temperature measurements and thermographic image acquisition, the sheep voluntarily entered self‐locking feeding stations while feeding, which minimised movement and ensured consistent positioning for image capture. Animals remained in the feeding stations for approximately 10 min before image acquisition to allow them to settle and minimise the influence of movement on thermographic measurements.

Immediately before each thermographic acquisition, RT was measured by a veterinary digital thermometer (Universal POD GmbH, Germany; REF/EAN 8031635916475; declared accuracy ±0.1°C) equipped with a flexible probe (≈10 s reading time). The probe was lubricated, inserted 4–6 cm into the rectum, and kept in place until stabilisation of the acoustic signal before a second reading was taken. The final value was calculated as the average of the two measurements. The probe was disinfected between animals following the manufacturer's instructions.

#### Hormonal verification of ovulation

In Sub‐trial 2, to quantify serum progesterone as a physiological marker of ovulation/luteinisation, blood samples were collected at 1715 h on Days 0–2 and Day 7 after sponge withdrawal (5 November 2024). Progesterone concentrations were measured by solid‐phase radioimmunoassay.^19^ Intra‐assay CVs were 6.91% and 8.71%, inter‐assay CV was 9.04% and assay sensitivity was 0.1 ng/mL. Values ≤0.5 ng/mL were classified as basal, and values >0.5 ng/mL indicated luteal activity.^20^ Rams were absent throughout the study.

### Data management and processing

Biologger data (°C) were downloaded by the Star‐Oddi communication box and Mercury v5.83 software (Star‐Oddi) and exported to a ‘.CSV’ file with UTC + 2 timestamps at 5‐min resolution. Rectal T (RT) readings were recorded in spreadsheets and consolidated into a CSV file (one row per measurement with exact time and animal ID). Infrared images captured with the FLIR E54‐EST camera were processed in FLIR Thermal Studio (Teledyne FLIR LLC, Wilsonville, OR, USA). For comparative purposes, maximum temperatures were extracted directly from the original thermographic images before FLIR Thermal Studio processing. These measurements are identified throughout the manuscript as ‘R’ (reference image) variables (VIRmax_R and AIRmax_R), whereas processed variables were obtained after ROI‐based analysis in FLIR Thermal Studio.

For IRT analysis, ROI were defined for the vulva and left axilla, with constant size and orientation per animal and session. From each ROI, mean *T* (*T*_mean_) and maximum *T* (*T*_max_) were extracted. Images showing blur, evident moisture/dirt or specular reflections were discarded. For biologger series, potential artefacts (defined as instantaneous rises or drops >0.8°C/min) were identified and excluded from the analyses. Imputation was not applied. RT readings were taken in duplicate and averaged.

An integrated dataset was assembled that contained the following eight *T* variables (all in °C, time‐stamped): subcutaneous T (SCT, biologger), rectal T (RT), the unprocessed hottest‐pixel maxima for vulva (VIRmax_R) and axilla (AIRmax_R) from direct images and the ROI‐based maxima and means for vulva (VIRmax, VIRmean) and axilla (AIRmax, AIRmean). Pairing *T*_max_/*T*_mean_ per peripheral site allowed comparisons between cutaneous signals and central measurements (biologger/rectal) and evaluation of how ROI standardisation (processed *vs*. unprocessed) affected thermal estimates.

To enable the mixed‐effects analysis (see Section: Statistical analysis), the data were reshaped into long format such that each row corresponded to Animal (ID) × Occasion × Method. An Occasion was defined as a unique measurement event for a given animal, identified by its date‐time stamp, during which the various *T* measurements were acquired quasi‐simultaneously. The long dataset included ID, Occasion, Reproductive stage (5 levels: Prepubertal, Pubertal, Sponge, Post‐sponge, Oestrous), Method (8 levels: RT, SCT, VIRmax_R, VIRmax, VIRmean, AIRmax_R, AIRmax, AIRmean) and Temperature (°C). If multiple recordings existed for the same ID‐Occasion‐Method, values were averaged to maintain one observation per cell. Missing values were kept as missing (no imputation). Categorical variables were encoded in a predefined order (physiological progression for stage, methodological hierarchy for method).

### Statistical analysis

All analyses were performed in IBM SPSS Statistics, version 26.0 (IBM Corp., Armonk, NY, USA). Body *T* (°C) was the dependent variable. We fitted a linear mixed‐effects model that included two fixed factors; viz., Method (8 levels: RT, SCT, VIRmax_R, VIRmax, VIRmean, AIRmax_R, AIRmax, AIRmean), Reproductive stage (5 levels: Prepubertal, Pubertal, Sponge, Post‐sponge, Oestrous) and their interaction (Method × Stage). A total of 2720 T records were available from repeated measurements on individually identified ewes across multiple occasions. Per‐factor sample sizes are reported in the Results.

To reflect the repeated and hierarchical structure of the data, the model included a random intercept for animal (ID) and a repeated term accounting for the correlation among methods measured within the same occasion; method‐specific variability was also allowed. Fixed effects were assessed using mixed‐model F‐tests. When significant effects or interactions were detected, estimated marginal means (EMMEANS) were compared using Sidak‐adjusted post‐hoc tests. For the Method × Stage interaction, simple‐effects comparisons were performed to evaluate differences among methods within each stage and differences among stages within each method. EMMEANS are reported with 95% confidence intervals or standard errors, as appropriate. Superscript letters in the tables indicate significant differences after Sidak adjustment (*p* < 0.05).

Model checking involved visual inspection of normal Q‐Q plots of residuals, residuals *vs*. fitted values (homoscedasticity) and boxplots of residuals by method. All available records were included in the analyses. Two‐sided tests were used and *α* = 0.05.

A mixed‐effects approach was chosen because measurements were repeated within animals and among methods on the same occasions, which created correlated and partially unbalanced data. A single mixed model provides coherent inference for main effects and their interaction without resorting to multiple separate univariate tests.

For the purposes of this study, precision was defined as the ability of a measurement method to consistently detect temperature differences associated with reproductive stage within animals. Agreement with core body temperature measurements (RT and SCT) was evaluated separately and is discussed in terms of comparability between methods rather than precision.

### Animal ethics statement

All animals were handled in accordance with Spanish animal welfare regulations (RD 1201/2005), aligned with European Union Directive 2010/63 on the protection of animals used for scientific purposes. The Ethical Committee on Animal Experimentation of the University of Salamanca reviewed and approved all procedures, issuing a favourable report under registration number 904.

## Results

### Serum progesterone verification

Serum progesterone profiles corroborated stage assignment because concentrations were basal around Days 1–2 after sponge withdrawal (mean ± SD = 0.48 ± 0.31 ng/mL) and increased markedly by Day 7 (mean ± SD = 3.05 ± 0.93 ng/mL), which confirmed luteal activity following induced oestrous (Table 1).

**Table 1:** Serum progesterone (ng/mL) in crossbred ewes after progestogen sponge withdrawal.

Sheep	Day 0	Day 1	Day 2	Day 7
1	2.25	1.39	<0.20	2.49
2	<0.20	0.27	<0.20	2.67
3	2.72	1.69	0.34	2.78
4	0.31	0.20	<0.20	4.71
5	0.30	<0.20	<0.20	2.60
Mean ± SD	1.15 ± 1.22	0.75 ± 0.73	0.22 ± 0.06	3.05 ± 0.93

### Overall model fit

We fitted a linear mixed‐effects model that included Method, Reproductive stage and their interaction as fixed effects, a random intercept for animal (ID) and a within‐occasion correlation across methods. Global fit indices confirmed the adequacy of the mixed model (−2 Restricted Log‐Likelihood [−2RLL] = 4610.66; Akaike Information Criterion [AIC] = 4684.66; Bayesian Information Criterion [BIC] = 4896.40). Those indices penalise unnecessary complexity; that is lower values indicate a better balance between fit and parsimony. The random‐intercept variance for animals was small and non‐significant (Wald *Z* = 1.30; p = 0.192), which suggested that, once Method, Stage and their interaction were accounted for, little residual heterogeneity remained at the ewe level. That indicated that the observed patterns were consistent among animals, rather than driven by a few outliers.

Within the repeated‐measures structure, variability was lowest for central measurements (RT, SCT), intermediate for vulvar thermography (VIRmax_R, VIRmax, VIRmean) and highest for axillary metrics (AIRmax_R, AIRmax, AIRmean), which matched the expected physiological pattern.

The long dataset comprised 2720 potential records, 2299 contributed valid residuals for diagnostics (421 missing, overall). The mixed‐model framework accommodates that unbalanced design without imputation and allowed coherent inference for main effects and their interaction.

### Global effects

In the mixed model, Method, Reproductive stage and their interaction all had significant (*p* < 0.001) effects (Table 2), which indicated that body temperature measurements differed among methods and reproductive stages, and that stage affected the differences between methods.

**Table 2:** F‐tests of fixed effects (measurement method, reproductive stage and their interaction) from the linear mixed‐effects model fitted to body temperature (°C) in crossbred ewes.

Effect	df	*F*	*p*
Method	7	378.126	<0.001
Reproductive stage	4	170.850	<0.001
Method × stage	28	35.416	<0.001

Abbreviation: df, degrees of freedom.

### Differences among measurement methods

EMMEANS indicated that RT detected the highest and AIRmean detected the lowest temperatures (Table 3). In addition, the 95% CIs suggested greater variability for the axillary infrared methods, particularly AIRmean, compared with the RT and SCT measurements, whereas the vulvar methods showed intermediate variability (Figure 2).

**Table 3:** Estimated marginal means (± SE) and 95% confidence intervals of body temperature (°C) derived from rectal, subcutaneous, vulvar infrared (processed and unprocessed) and axillary infrared (processed and unprocessed) measurement methods in crossbred ewes.

Method	Mean	SE	95% CI	*p*
RT	39.62^a^	0.051	39.48–39.77	<0.001
SCT	38.94^b^	0.059	38.78–39.10	<0.001
VIRmax_R	39.25^c^	0.068	39.06–39.44	<0.001
VIRmax	39.11^d^	0.068	38.92–39.30	<0.001
VIRmean	37.34^ef^	0.108	37.04–37.64	<0.001
AIRmax_R	37.25^e^	0.151	36.83–37.67	<0.001
AIRmax	37.64^f^	0.120	37.30–37.97	<0.001
AIRmean	32.97^g^	0.202	32.41–33.53	<0.001

Abbreviations: AIRmax, axillary infrared (FLIR), maximum; AIRmax_R, axillary infrared (reference camera), maximum; AIRmean, axillary infrared (FLIR), mean; CI, confidence interval; RT, rectal temperature; SCT, subcutaneous temperature (biologger); SE, standard error; VIRmax, vulvar infrared (FLIR), maximum; VIRmax_R, vulvar infrared (reference camera), maximum pixel temperature; VIRmean, vulvar infrared (FLIR), mean.

*Note*: Different superscript letters indicate significant differences among methods based on Sidak‐adjusted post‐hoc comparisons of estimated marginal means (*p* < 0.05).

**Figure 2:**
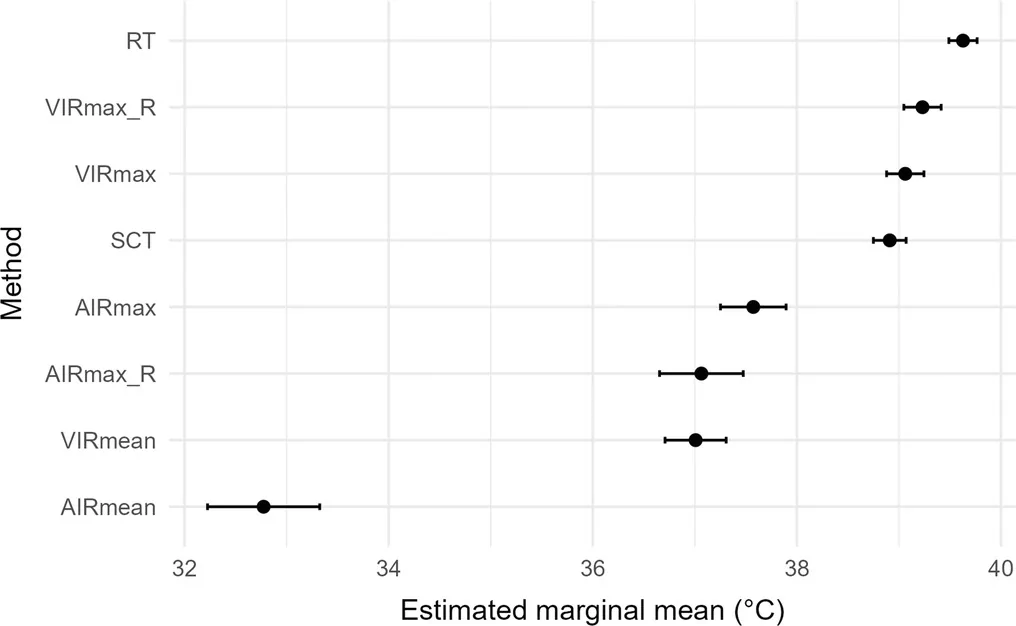
Measurement method and estimated marginal mean temperature in crossbred ewes (EMMs ±95% CI). AIRmax, axillary infrared (FLIR), maximum; AIRmax_R, axillary infrared (reference camera), maximum; AIRmean, axillary infrared (FLIR), mean; CI, confidence interval; RT, rectal temperature; SCT, subcutaneous temperature (biologger); VIRmax, vulvar infrared (FLIR), maximum; VIRmax_R, vulvar infrared (reference camera), maximum pixel temperature; VIRmean, vulvar infrared (FLIR), mean.

### Method × stage interaction

The significant Method × Stage interaction effect (Table 4, Figure 3) indicated that the magnitude of temperature changes differed among measurement methods across the age‐ and treatment‐related stages evaluated. Overall, rectal temperature (RT) and subcutaneous temperature (SCT), which represent core body temperature, showed the most consistent patterns throughout the study.

**Table 4:** Estimated marginal means (± SE) of body temperature (°C) showing the interaction between measurement method (rectal, subcutaneous, vulvar infrared and axillary infrared) and age or treatment stage in crossbred ewes.

Age stage			Treatment stage			
Method	Prepubertal	Pubertal	Sponge	Post‐sponge	Oestrous	*p*
RT	40.09 ± 0.055^a^	39.74 ± 0.061^b^	39.53 ± 0.052^c^	39.39 ± 0.061^d^	39.37 ± 0.072^d^	<0.001
SCT	39.46 ± 0.073^a^	38.98 ± 0.098^b^	38.80 ± 0.065^b^	38.76 ± 0.089^b^	38.67 ± 0.117^b^	<0.001
VIRmax_R	40.23 ± 0.091^a^	39.48 ± 0.118^b^	38.88 ± 0.073^c^	38.68 ± 0.122^c^	38.99 ± 0.160^bc^	<0.001
VIRmax	40.17 ± 0.091^a^	39.30 ± 0.118^b^	38.68 ± 0.073^c^	38.48 ± 0.121^c^	38.93 ± 0.160^bc^	<0.001
VIRmean	39.23 ± 0.160^a^	38.24 ± 0.220^b^	35.45 ± 0.127^c^	36.20 ± 0.243^d^	37.58 ± 0.311^b^	<0.001
AIRmax_R	39.70 ± 0.026^a^	37.32 ± 0.320^b^	35.74 ± 0.186^c^	37.18 ± 0.333^b^	36.29 ± 0.476^bc^	<0.001
AIRmax	39.68 ± 0.178^a^	37.71 ± 0.250^b^	36.41 ± 0.150^c^	37.33 ± 0.251^b^	37.06 ± 0.364^bc^	<0.001
AIRmean	38.08 ± 0.306^a^	34.86 ± 0.430^b^	29.18 ± 0.283^c^	31.63 ± 0.447^d^	31.10 ± 0.644^cd^	<0.001

Abbreviations: AIRmax, axillary infrared (FLIR), maximum; AIRmax_R, axillary infrared (reference camera), maximum; AIRmean, axillary infrared (FLIR), mean; RT, rectal temperature; SCT, subcutaneous temperature (biologger); SE, standard error; VIRmax, vulvar infrared (FLIR), maximum; VIRmax_R, vulvar infrared (reference camera), maximum pixel temperature; VIRmean, vulvar infrared (FLIR), mean.

*Note*: Within each row, different superscript letters indicate significant differences among reproductive stages for that method, based on Sidak‐adjusted post‐hoc comparisons (*p* < 0.05). Letters should not be compared between rows.

**Figure 3:**
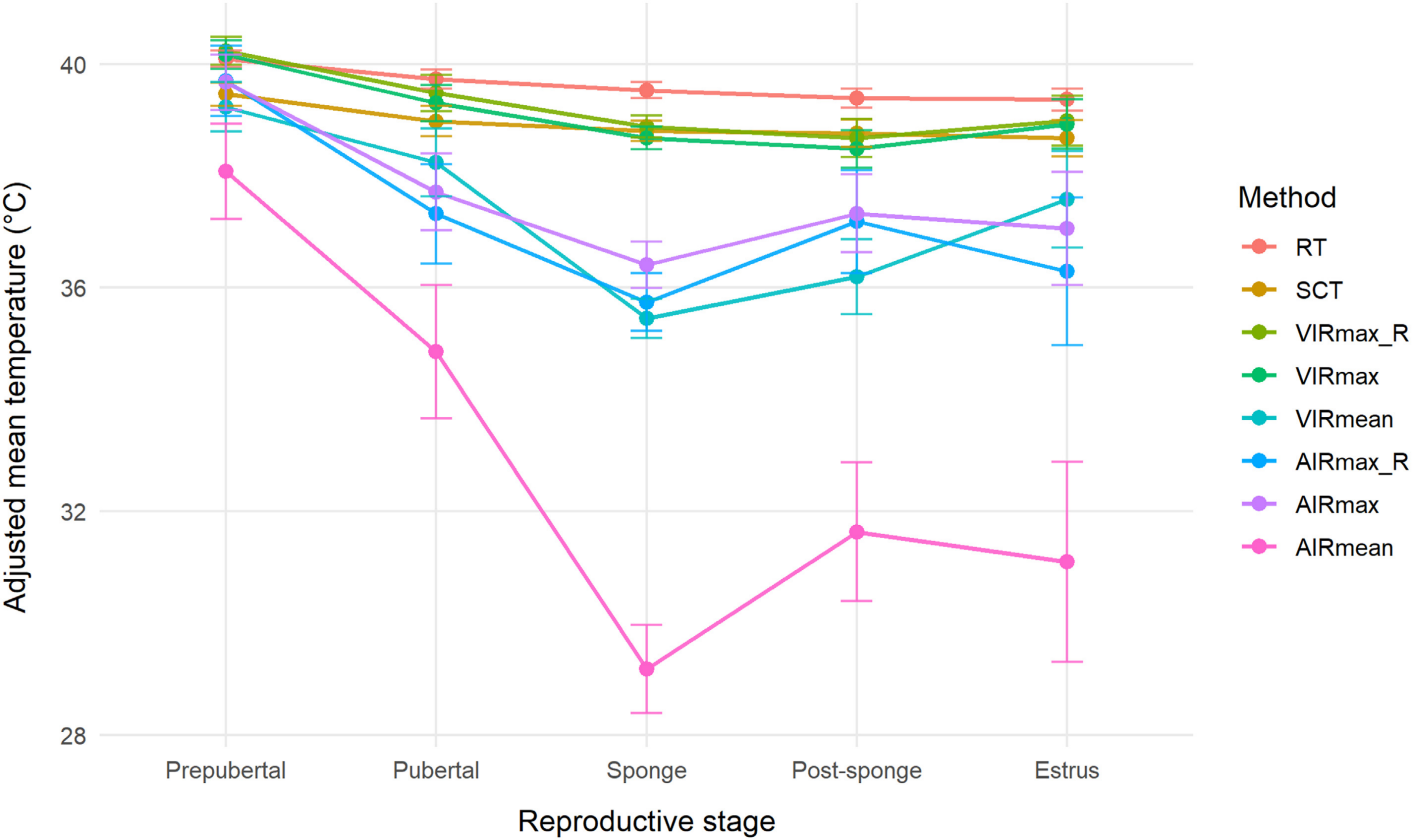
Method × stage interaction effect on body temperature (°C) measurements in crossbred ewes. AIRmax, axillary infrared (FLIR), maximum; AIRmax_R, axillary infrared (reference camera), maximum; AIRmean, axillary infrared (FLIR), mean; RT, rectal temperature; SCT, subcutaneous temperature (biologger); VIRmax, vulvar infrared (FLIR), maximum; VIRmax_R, vulvar infrared (reference camera), maximum pixel temperature; VIRmean, vulvar infrared (FLIR), mean.

Both RT and SCT progressively decreased from the prepubertal stage to the later treatment stages. Rectal temperature declined from 40.09 ± 0.055°C in prepubertal ewe lambs to 39.36 ± 0.072°C in the expected oestrous period, while SCT decreased from 39.46 ± 0.073°C to 38.67 ± 0.117°C over the same period. These results suggest that both increasing age (prepubertal to pubertal transition) and hormonal treatment were associated with a gradual reduction in core body temperature.

The lowest core temperatures were observed during the late treatment stages (post‐sponge and oestrous) for both RT and SCT, indicating that the combined effects of maturation and reproductive treatment resulted in sustained reductions in body temperature during the final stages of the study. Vulvar infrared measurements showed a similar overall tendency, although with greater variability, whereas axillary measurements remained the lowest and most variable throughout all stages.

Figure 3 illustrates these stage‐dependent changes, highlighting the progressive decline in rectal and subcutaneous temperatures from the prepubertal stage to the expected oestrous stage.

## Discussion

Our study confirmed that body *T* in ewes varied systematically among reproductive stages, and that the anatomical window and method of measurement had a significant effect on the magnitude and reliability of these fluctuations. Recent evidence has indicated that patterns in body temperature in small ruminants follow stage‐specific physiological dynamics, and measurement accuracy differs substantially among sensor types.^21^, ^22^ In our study, the mixed‐model EMMEANS indicated that RT was the method that provided the highest mean values, AIRmean provided the lowest and the values from SCT and vulvar thermography measurements were intermediate (VIRmax_R, VIRmax, VIRmean). Furthermore, the significant Method × Stage interaction effect indicated that those differences were not constant among physiological phases, a pattern that has been reported in studies of endocrine‐driven thermoregulation.^23^

Physiologically, the highest temperatures occurred in the prepubertal stage, followed by a progressive decline across the subsequent reproductive stages. However, the magnitude and timing of these thermal changes differed among measurement methods, which reflects the endocrine modulation of thermoregulation in small ruminants.^23^ Progesteronaemia can suppress central and peripheral T, but the peri‐oestrous phase involves a moderate temperature increase, which is associated with an increase in blood flow and metabolic activity in reproductive tissues.^21^ The results of our study, together with established relationships between endocrine activity and thermoregulation, indicate that *T* is a reliable physiological indicator of reproductive status.

Methodologically, central windows (RT and SCT) exhibited the lowest residual variance and the narrowest confidence intervals, confirming their greater stability, comparable to the precision of implanted or core‐temperature sensors in ruminants.^22^, ^24^ Peripheral axillary measurements exhibited the highest variability, and environmental exposure, fleece thickness and skin conductance can significantly reduce the stability of superficial thermal readings.^25^ Within peripheral regions, vulvar thermography provided the most consistent detection of reproductive‐stage thermal changes, and previous studies have suggested that anatomical windows that have high vascularisation and low insulation are preferable for non‐invasive infrared imaging.^21^, ^25^

Analytically, in our study, the linear mixed‐effects approach was essential for accommodating the hierarchical structure (repeated measures within animal), unbalanced cells among methods and stages and cross‐method dependence within occasions. Similar modelling strategies have been recommended for studies that involve multi‐sensor reproductive monitoring because of their robustness in handling hierarchical, irregular and physiologically correlated data.^24^ The robust global effects, coherent interaction effects and well‐behaved diagnostics support the validity of this framework in multi‐sensor reproductive monitoring.

Beyond methodological considerations, our study reflects the applied value of biologgers in reproductive management. Continuous, high‐resolution T data can detect circadian rhythms, behavioural transitions and endocrine changes with high sensitivity,^24^ which reflects their potential in detecting oestrous early. Recently developed integrative systems such as ReproBehaviorNet, which combine physiological, behavioural and thermal variables in deep‐learning algorithms, reflect the growth of the integration of multi‐modal sensors in precision livestock farming.^26^ Such hybrid AI‐based approaches are similar to the monitoring architecture used in our study, and might substantially enhance the early, minimally invasive detection of key reproductive events in sheep.^21^, ^22^, ^23^, ^24^, ^26^

From a practical perspective, the applicability of these findings may extend to extensive sheep production systems. The present study was conducted in a semi‐extensive Mediterranean production system located in the Spanish dehesa, a traditional agro‐silvopastoral ecosystem characterised by low stocking densities, extensive grazing, natural pastures and scattered oak trees.^27^ Although not identical, this production system shares several functional and management characteristics with many Australian rangelands production systems, including a strong dependence on natural resources, marked seasonal environmental variability and low‐input livestock management.^27^, ^28^ Under these conditions, frequent animal handling for reproductive monitoring can be labour‐intensive, costly and potentially stressful for animals. Therefore, although RT and SCT provided the most reliable detection of temperature changes associated with reproductive stage, vulvar IRT may offer the most practical non‐invasive alternative for extensive production systems.

Beyond reproductive monitoring, these approaches may also have value for the early detection of puberty in replacement ewe lambs and for studies investigating thermoregulation and heat‐stress responses in sheep under commercial production conditions.^29^

In general, our study has confirmed that body T can be a sensitive, biologically meaningful biomarker of reproductive status in ewes if the appropriate anatomical window and sensor technology are chosen. Our results, which were very similar to other thermal, endocrinological and sensor‐based research,^21^, ^22^, ^23^, ^24^, ^26^ argue for the development of more efficient, ethical and scalable precision‐livestock strategies for reproductive monitoring in small ruminants.

## Conclusions

Body T was a useful biomarker of reproductive status in ewes. RT and SCT provided the most stable and sensitive detection of temperature changes associated with reproductive stage, whereas vulvar IRT emerged as the most promising non‐invasive alternative. In contrast, axillary measurements showed greater variability and lower precision. The integration of SCT monitoring and vulvar IRT with machine‐learning approaches may provide the basis for future automated reproductive monitoring and decision‐support systems in precision sheep farming.

## Conflicts of interest and sources of funding

The authors declare no conflicts of interest for the work presented here. The study was supported by a 2024 Leonardo Grant for Scientific Research and Cultural Creation, Fundación BBVA (LEO24‐1‐13,708‐ING‐ING‐119).

## Data Availability

The data that support the findings of this study are available from the corresponding author upon reasonable request.
